# Oil Effect on Improving Cracking Resistance of SBSMA and Correlations Among Performance-Related Parameters of Binders and Mixtures

**DOI:** 10.3390/ma18235443

**Published:** 2025-12-03

**Authors:** Ronghua Gu, Jing Xu, Weihua Wan, Kai Zhang, Yaoting Zhu, Xiaoyong Tan

**Affiliations:** 1 Jiangxi Transportation Engineering Group Co., Ltd., Nanchang 330000, China; 99308701@163.com; 2 Jiangxi Provincial Highway Engineering Co., Ltd., Nanchang 330006, China; 3 School of Civil Engineering and Architecture, East China Jiaotong University, Nanchang 330013, China; jingxv@ecjtu.edu.cn; 4 Jiangxi Communications Investment Maintenance Technology Group Co., Ltd., Nanchang 330052, China; kaizhang@whut.edu.cn (K.Z.); 15289471406@163.com (Y.Z.); tanxy@ecjtu.edu.cn (X.T.)

**Keywords:** oil effect, SBSMA, cracking resistance, cracking performance parameters, aging, correlations

## Abstract

Asphalt binders that perform exceptionally well in resisting both rutting and cracking are highly desirable for withstanding the combined effects of extreme low temperatures and heavy vehicle loads. This work highlights the benefits of softening oils on the cracking performance of styrene–butadiene–styrene-modified asphalt (SBSMA). Additionally, the inherent correlations between cracking-performance parameters of binders and mixtures were thoroughly analyzed. A bio-based oil (bio-oil) and a petroleum-based oil (re-refined engine oil bottom, REOB) were selected as the softening oils. The benefit provided by softening oils was evaluated using various rheological indices, while the adverse effects of oxidative aging on cracking resistance were also considered. The cracking properties at intermediate temperatures were characterized by the modified Glover–Rowe (M G–R) parameter, δ_8967 kPa_, and fatigue life (N_f_). The low-temperature cracking properties of binders were evaluated by stiffness and m-value. The indirect tensile asphalt cracking (IDEAL-CT) test was conducted utilizing the CT-index and post-peak slope to estimate the fracture properties of the mixtures. The oxidative aging of binder and mixture samples was simulated and carried out based on lab aging methods; meanwhile, the carbonyl index obtained from the Fourier transform infrared (FTIR) scanning was used to track and evaluate the aging level of binders. The results show that the cracking performance could be greatly improved by the application of softening oils. Meanwhile, the bio-oils were found to operate with much higher efficiency than REOB, since the oil modification index (OMI) result showed that bio-oils exhibited four to six times the efficiency of REOB, in terms of improving the stress relaxation property. The correlations proved that the cracking-related parameters shared an inherent relationship with R^2^ above 0.85, while these parameters consistently declined as the binder aged. The cracking performance of the mixtures at intermediate temperatures was mainly governed by the fatigue life of the binder, whereas thermal cracking performance was highly associated with the binder’s relaxation property.

## 1. Introduction

SBS polymer modification on asphalt is a desirable technology for pavement binding materials, since it can effectively enhance the elastic response and greatly improve the creep recovery property of binders, which enables pavement binders to resist permanent deformation and rutting at high temperatures and to carry the rising traffic volumes and vehicle loads [1,2]. This enhancement can be attributed to the three-dimensional networks formed by SBS polymers at the appropriate dosage [3]. The SBS polymer is a typical triblock copolymer, exhibiting a two-phase morphology consisting of polystyrene domains linked by polybutadiene segments [4]. PS is glassy, enhancing the hardness of the binders, while PB is rubbery, improving the elastic response and creep recovery. Recently, high-polymer-modified asphalt, often used in the upper layer, permeable asphalt concrete, etc., has been produced by the application of high dosage SBS (e.g., 7% or above), where the high content of SBS provides extra viscosity and binding strength [5,6].

Oxidation aging is inevitable for paving asphalt binders, occurring at the early stage (e.g., mixing, transporting, compacting, etc.) and the service period that corresponds to short- and long-term aging, respectively [7]. Nevertheless, aging of SBSMA consists of not only aging of binders, which results in an overall increase in stiffness and viscosity (age hardening), but also the degradation of SBS polymers, which can exhibit a softening effect and break the SBS networks [8,9]. Research results have shown that short-term aging (normally at the initial stage) may temporarily decrease the rutting resistance of SBSMA, which is primarily attributed to SBS degradation (the softening effect), and offset the age hardening of binders [10]. Under environmental factors like oxygen, heat, ultraviolet light, etc., the tendency of SBS polymers to degrade mainly originates from the nature of the chemical compositions: the substantial double-carbon bonds within the SBS molecule are unsaturated and chemically unstable [11,12]. Thus, the long-term performance and the impact of oxidation aging should also be carefully considered.

During long-term service, thermal cracking is one of the most common distresses that asphalt pavements face. Generally, SBSMA binders/mixtures underperform at low temperatures. In the performance grade (PG) system, the improvement in low-temperature (LT) PG is not as great as that in high-temperature (HT) PG after SBS modification of the neat binder [13,14,15]. Additionally, in some special areas (e.g., Xinjiang region of China), where there is a harsh environment of both extreme high and low temperatures, binders with high hardness to withstand rutting and great relaxation to avoid thermal cracking are desired. Kabir’s research showed that a balanced combination of SBS and softening agent (suitable type and dosage) could reach an overall improvement in performance, in which resistance to cracking and anti-rutting performance were elevated simultaneously [16]. Additionally, Issa found that bio-based oil exhibited higher efficiency than paraffinic oil in promoting low- and intermediate-temperature cracking performance [17]. It was also confirmed that high-polymer-modified asphalt binders incorporating softening oils were capable of excellent performance regarding heavy traffic loads and extreme cold weather conditions [18,19].

To characterize the cracking performance of neat and modified asphalt binders, a number of parameters were developed and proposed. In the PG system, parameters including fatigue factors and stiffness as well as m-value are used in conjunction with fatigue and thermal cracking resistance within the linear viscoelastic range (low strain levels) [20]. The linear amplitude sweep (LAS), concluded in the PG plus system, applies up to a 30% strain level to detect the failure behavior of binders; meanwhile, the viscoelastic continuum damage (VECD) principle is employed to calculate the fatigue life of binders at the applied strain level [21,22]. Zhang and Bahia found that the fatigue life of binders obtained from LAS at relatively high strain levels (i.e., 10% strain) displayed a fair correlation with mixtures [23]. Similarly, single edge notched beam (SENB) was developed to capture the low-temperature fracture behaviors and cracking properties of binders, in which a notch was designed in the middle of the beam to ensure a fracture occurs during testing [24]. Other rheological indices, like δ_8967 kPa_ and ΔTc, were also utilized to estimate the cracking performance from different perspectives [25,26].

To conclude, there is a possibility and need to obtain a good combination of SBS polymer and softening oils to reach a highly improved cracking performance while maintaining excellent HT rutting resistance. In this work, a bio-based oil (corn oil) and a petroleum-based oil (re-refined engine oil bottom, REOB) were selected as the softening oils. Besides the PG grading system, various rheological indices were employed to estimate the benefit of the addition of the oils into SBSMA in regards to cracking performance. The indirect tensile asphalt cracking (IDEAL-CT) test was conducted to estimate the fracture properties of the mixtures. Meanwhile, the influence of oxidation aging was also considered, and the oxidation level of the binders was tracked by Fourier transform infrared (FTIR) spectroscopy. The main objectives of this work are summarized as follows:

(1) Investigate the oil effect on improving the cracking performance of SBSMA and consider the impact of oxidation aging;
(2) Explore the internal relationship among cracking performance parameters of the binders;
(3) Correlate the performance-related indices between the binder and mixture and identify the binder parameters that can be used to predict the mixture cracking performance.

## 2. Materials and Methods

### 2.1. Materials and Preparation of Specimens

#### 2.1.1. Binder Materials and Preparation of Specimens

A base binder (PG58-28) was used to prepare all binder and mixture samples. There are two selected SBS polymer modifiers: Dushanzi T6302H produced in China and LG 501 produced in Korea. The molecular structure of both SBS polymers is linear with a styrene–butadiene ratio of 30/70. The tensile strengths of the Dushanzi T6302H and LG 501 SBS modifiers are 20 and 26 MPa, respectively.

One bio-based oil (bio-oil) derived from corn and one petroleum-based oil (re-refined engine oil bottom, REOB) were used to improve the cracking performance of SBSMA, with the fundamental information of the softening oils presented in Table 1.

The preparing procedure of the SBSMA samples included three steps [4,13]: first, the base binder was heated to 180 °C, after which, the SBS and sulfur at the determined dose were carefully added with the mixer at a low rate (700 rpm) for 30 min; then, the residues were shifted from the mixer to the shearer with a rate of 5000 rpm at 180 °C for 1 h; lastly, the residues were conditioned in an oven at 170 °C for 30 min. The oil/SBS-modified asphalt was prepared by the addition of oil into the SBSMA under a low mixing rate of 700 rpm for 30 min. The addition of sulfur into the SBSMA aimed to improve storage stability [8,13]. The exact compositions of the binder samples and the corresponding description are shown in Table 2.

HT and LT correspond to high and low temperature. Notice that the material compositions (the dosage of oil and SBS) for each sample may vary to ensure that the oil/SBS-composite-modified asphalt sample reaches the same PG (PG76-34).

#### 2.1.2. Mixture Materials

The mix design selected for this work is a typical surface layer used in China’s highways, and all mixture samples were prepared with the same mix gradation, as shown in Table 3. All mixture samples were compacted in a Superpave Gyratory Compactor for 75 gyrations to ensure constant volumetric properties.

Notice that the binder content is a weight percentage of asphalt binder compared with the total aggregates.

### 2.2. Aging Method

In this study, the binder samples, including the base and oil/SBS-modified asphalt binders, were exposed to oxidation aging, of which short-term aging was achieved by a rolling thin film oven (RTFO) at 163 °C for 75 min, with regard to AASHTOT 240, and long-term aging was simulated by a 20 and 40 h pressure aging vessel (PAV20 and PAV40), following AASHTO R 28.

The mixture samples were only subjected to long-term aging to study the aging effect on cracking performance. According to AASHTO R 30 [31] specifications, conditioning the loose mixtures in an oven at 135 °C for 4 h is the standard procedure to simulate short-term aging. This study simulated long-term aging by conditioning the loose mixtures in an oven at 135 °C for 8 h, following the method developed by the Wisconsin Highway Research Program (WHRP) 17-04 [32,33].

### 2.3. Binder Experiments and Indices

#### 2.3.1. Bending Beam Rheometer (BBR) Tests

Low-temperature performance grading was conducted on a bending beam rheometer (BBR) with 20 h PAV-aged samples at −18 °C and −24 °C, where the stiffness and m-value at 60 s were selected. The continuous LT PG grade was calculated by the linear interpolation method when the stiffness reached 300 MPa or m-value reached 0.3. Additionally, it has been proven that there is a linear relationship between oil content and BBR parameters [34]. So, the oil modification index (OMI) was proposed to estimate the oil efficiency on softening binders and improving the inner stress relaxation property, and it can be calculated based on Equation (1):

(1) $$OMI\;=\;\frac{The\;change\;of\;BBR\;parameter}{oil\;content}\;$$

where the BBR parameter refers to the logarithm of stiffness or m-value. The change in the PG parameter is equal to the PG parameter after adding oil minus the PG parameter before adding oil. The OMI represents the change in the log (stiffness) or m-value for a 1% addition of oil.

#### 2.3.2. Linear Amplitude Sweep (LAS) Tests

The LAS test was developed and proposed in the PG plus grading system to detect the resistance to fatigue cracking of binders at intermediate temperature, in which the testing time is much shorter than that of the traditional method [21,22]. The whole LAS testing consisted of two main parts: a frequency sweep that was conducted at a 0.1% strain level (within the linear viscoelastic range) to characterize the binder properties at the undamaged state and an amplitude sweep with the strain level from 0.1% to 30% at a constant frequency of 10 Hz to quickly accumulate binder damage. For comparison purposes, all LAS tests were carried out at the same temperature of 25 °C, where the cycles to failure (N_f_) at a certain strain level (γ) could be obtained by Equation (2), in accordance with AASHTO TP 101-14, and at least two replicates were tested for one sample:

(2) $$N_{f}=A{\left(\gamma_{max}\right)}^{B}$$

where parameter A and B refer to the model coefficient determined by the characteristics of the materials. Parameter A can be simply regarded as N_f_ at a strain level of 1%, while parameter B is the slope of the logarithm of N_f_ versus the logarithm of the applied strain.

Besides N_f_, the modified Glover–Rowe (M G–R) parameter can be derived from the frequency sweep of the LAS tests, with the calculation based on Equation (3), while G–R originally was proposed to indicate the cracking performance of binders, with the corresponding complex shear modulus ($G^{*}$) and phase angle (δ) selected from the corresponding master curves at 15 °C and 0.005 rad/s [15]:

(3) $$M\;G\;-\;R=\frac{{G^{*}\left[\cos\left(\delta \right)\right]}^{2}}{\sin\left(\delta \right)}\;$$

where $G^{*}$ and δ are selected in the frequency sweep of the LAS tests as the frequency reaches 10 rad/s. The unit of M G–R is Pa.

#### 2.3.3. Frequency Sweep Tests and δ_8967 kPa_

To obtain the phase angle δ_8967 kPa_ when G* is equal to 8967 kPa, several frequency sweeps were conducted, with the frequency increasing from 1 to 30 rad/s at 0, 10, 18, and 25 °C. Notice that the applied strain level should ensure that all samples stay within the linear viscoelastic range. The calculating method of the phase angle δ_8967 kPa_ includes the following: first, select the G* and δ for all testing temperatures (0, 10, 18, and 25 °C) when the frequency is 10 rad/s (1.59 Hz); then, calculate the temperature (G* = 8967 kPa) with the linear interpolation (temperature vs. log G*) using the selected G* and δ; lastly, the δ_8967 kPa_ for each sample can be calculated by the linear interpolation (temperature vs. phase angle). A binder with a higher δ_8967 kPa_ indicates a better cracking performance [26].

### 2.4. ATR-FTIR Scanning

A Bruker TENSOR and the attenuated total reflection (ATR) Fourier transform infrared spectroscopy (ATR-FTIR) method were used to measure the spectra of each binder at every aging condition. The spectra were obtained by an average of 32 spectra at 2 cm^−1^ resolution, with the wavenumber range of 400–4000 cm^−1^. The baseline correction and band normalization were carried out using professional software (Thermo Scientific OMNICTM version 8.0), after which, the quantitative analysis on functional groups was conducted using MATLAB version 2016 software.

**Figure 1:**
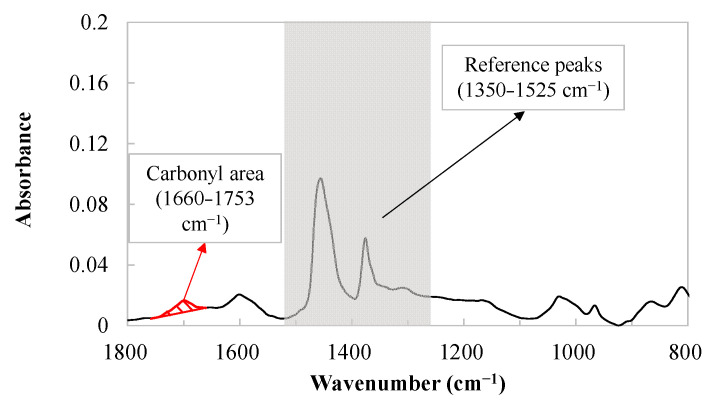
The typical ATR-FTIR spectra of asphalt binders.

Carbonyl and sulfoxide are the easily distinguished oxygen-containing functional groups and often used as indices to assess the aging degree of binders [35]. However, it has been proven that sulfoxide is not chemically stable and tends to degrade at high temperatures [35]. So, the carbonyl index was selected to indicate the aging level of the binder samples, with the carbonyl index defined as follows:

(4) $$Carbonyl\;index=\frac{Carbonyl\;area\;}{Reference\;peaks\;area}$$

where the carbonyl area and reference peaks area were fixed at the ranges of 1660–1753 and 1350–1525 cm^−1^, respectively, seen in Figure 1. The carbonyl area was measured in arbitrary units, as a surrogate of binder oxidation level.

### 2.5. Mixture Experiments and Indices

**Figure 2:**
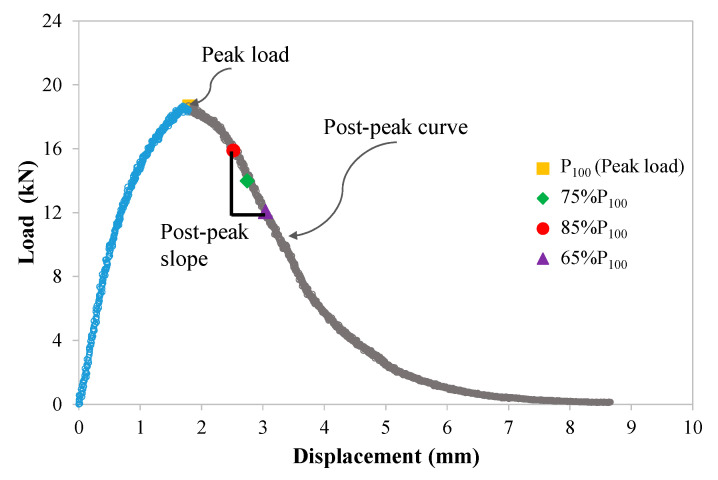
The representative load–displacement curve of IDEAL-CT test.

The mixture fatigue and thermal cracking performance was assessed by indirect tensile asphalt cracking (IDEAL-CT) tests at 25 °C and 0 °C, respectively, with the testing procedure following ASTM D8225-19. This experiment was conducted at a constant loading rate of 50 mm/min. The load was applied and controlled by a servo-hydraulic loading device. A representative load–displacement curve at low temperatures measured in the IDEAL-CT test is presented in Figure 2.

From the curve, fracture energy (G_f_) and post-peak slope can be used to precisely characterize mixture cracking properties [36] and can be derived using Equation (5):

(5) $$CT-index=\frac{t}{62}\times \frac{l_{75}}{D}\times \frac{G_{f}}{\left|m\right|}\times 10^{6}$$

where G_f_ is the whole fracture energy during the IDEAL testing and $\left|m\right|$ is the slope of the post-peak curve where the load reduces to 75% of the peak load (PPP75). The post-peak slope in this study is equal to the absolute value of $\left|m\right|$; l75 represents the displacement of the post-peak curve where the load decreases to 85% of the peak load; D is the diameter of the testing samples.

## 3. Results and Discussion

### 3.1. Low-Temperature Cracking Parameters from BBR Tests

**Figure 3:**
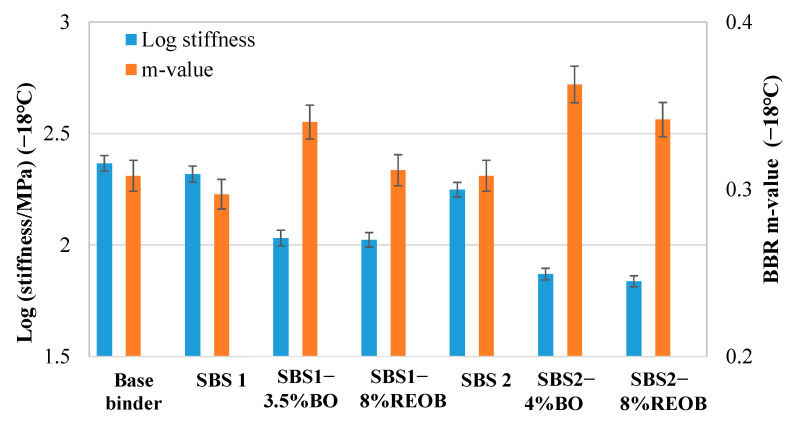
The stiffness and m-value results from BBR tests.

In the performance grade (PG) grading system, the LT cracking performance is determined by both stiffness and m-value. The LT cracking performance of modified binder samples that have been subjected to PAV aging was evaluated by the BBR test, with the logarithm of stiffness and m-value results shown in Figure 3.

Unlike the HT rutting performance, the LT cracking performance of SBSMA was not greatly improved: the stiffness and m-value of the SBS1 and SBS2 samples did not decrease and elevate, respectively, as compared to those of the base binder. It can be clearly seen that there was a consistent trend in both LT PG grading figures, where the logarithm of stiffness went down and m-value went up greatly, after the addition of oils into the SBSMA samples.

Additionally, it was found that the oil effect on the LT cracking performance of SBSMA also varied with the oil type: the bio-oil highly outperformed the REOB. The m-value of the samples incorporating bio-oil was significantly higher than that of the samples with REOB, implying the bio-oil works with higher efficiency with regard to enhancing the relaxation property. The values of the logarithm of stiffness for all oil–SBS-modified asphalt binders were very close, indicating a similar oil effect in terms of softening the binders (decreasing the stiffness).

The OMI obtained from the logarithm of stiffness is inherently a negative value, with a lower value exhibiting a higher efficiency in softening the binders, which can be regarded as benefiting cracking performance [37,38]. On the other hand, the OMI derived from the m-value is a positive value: a higher value means a better efficiency in improving the inner stress relaxation property. The OMI results obtained from the logarithm of stiffness and m-value are presented in Table 4.

We found that the OMI results are consistent with the stiffness and m-value results. The bio-oil operated with twice the efficiency of REOB with regard to softening the binders and four to six times the efficiency of REOB in terms of improving the stress relaxation property. This also confirms that the dosage of REOB normally could be much higher than that of bio-oil when pursuing the similar oil effect (i.e., the similar reduction in LT PG).

### 3.2. Black Diagrams and M G–R Results

The original G–R parameter (15 °C and 0.005 rad/s) has been proven to be highly correlated to ductility and can be used as an indicator of cracking performance. In the original black diagrams, there are two damage curves at the brittle rheological behavior: one at G–R equal to 180 kPa, implying damage onset, and the other at 600 kPa, indicating significant cracking. Yet, this established threshold cannot be directly applied in M G–R, since the G* and δ of M G–R were captured at 25 °C and 10 rad/s. Therefore, black space diagrams were plotted (showing G* versus phase angle at 25 °C and 10 rad/s); meanwhile, M G–R results were derived mainly for comparison.

**Figure 4:**
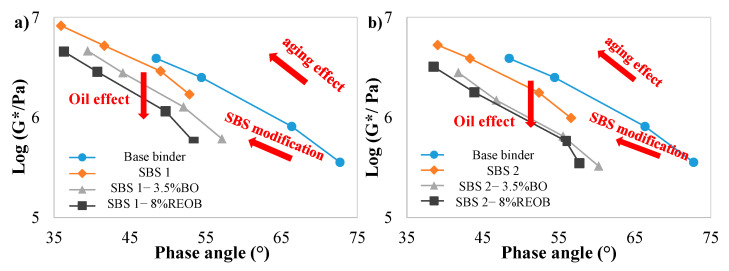
Black spaces at each aging state: (**a**) G* and δ at 25 °C; (**b**) G* and δ at 10 rad/s.

Figure 4 presents the G* and δ at 25 °C and 10 rad/s in the black diagrams, and there are four markers for each binder sample representing four aging states: unaged, RTFO, PAV20, and PAV40. It was clear that the aging effect moved the marker of each sample from the bottom-right to the upper-left in the figure.

What is interesting in these black diagrams is that the aging effect is similar to the SBS modification, since both effects caused a similar trend on the curve: moving from the bottom-right to the upper-left (approaching cracking).

On the other hand, the incorporation of oil into SBSMA exhibited an opposite effect on the curve in the black diagram, where the curve shifted downwards in the graph after adding oils. A tiny distinction in the moving direction of the curves between adding bio-oil and REOB was found: toward the bottom-right and straight down. This indicates that adding bio-oil was capable of not only reducing the complex modulus (softening effect) but also enhancing the viscous response (phase angle), while REOB only exhibited the softening effect. It also confirms the conclusion that bio-oil outperformed REOB, with regrading improving resistance to cracking.

**Figure 5:**
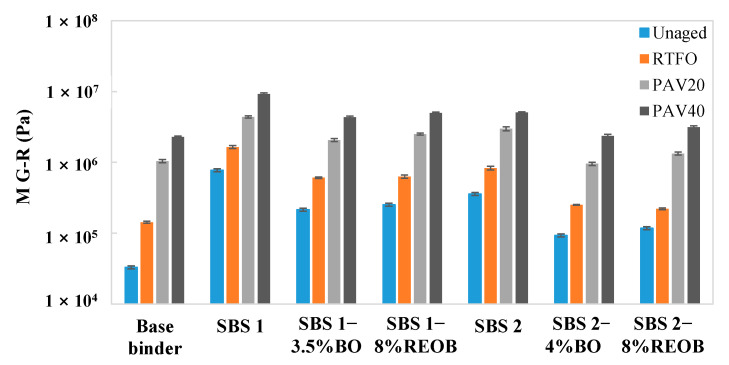
The M G–R results from the frequency sweep of LAS tests at each aging state.

An M G–R with a higher value could be viewed as detrimental to the binder’s resistance to cracking. From Figure 5, it is acknowledged that the SBS modification on the binder caused a significant increase in M G–R, since the M G–R value of SBS 1 and SBS 2 is greatly larger than that of the base binder. This may denote that the SBS modification does not benefit the binder in terms of the intermediate-temperature cracking performance.

When comparing M G–R before and after adding oil, an obvious downward trend is observed, while the reduction brought in by bio-oil is relatively larger than that by REOB. However, when looking at the M G–R of the binders at aging conditions of PAV20 and 40, the values of the samples with and without oils are very close. This indicates that the oil effects on improving cracking performance would diminish after long-term aging.

### 3.3. δ_8967 kPa_ Results

The oil effect on the binder’s resistance to thermal cracking was further characterized with the employment of δ_8967 kPa_ that was derived from the requirement on the intermediate-temperature fatigue factor less than 5000 kPa in the PG system. A binder with a higher δ_8967 kPa_ can be regarded as exhibiting a better relaxation ability and a greater fatigue cracking resistance.

**Figure 6:**
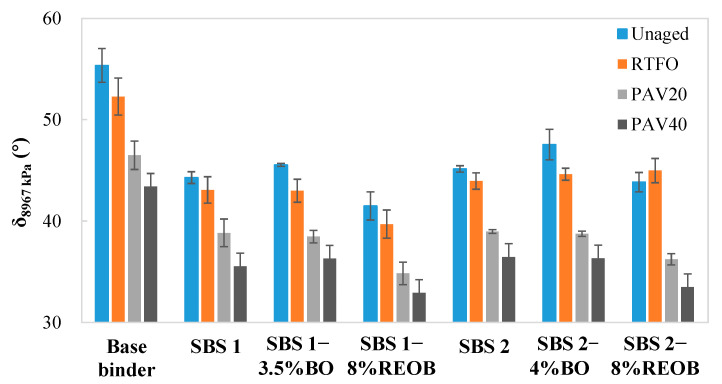
The δ_8967 kPa_ results at each aging state.

Figure 6 compares the δ_8967 kPa_ results of binder specimens with variable compositions at different aging conditions. First, it is easily realized that the SBS modification resulted in a significant drop in the δ_8967 kPa_, indicating the base binder exhibits an advantage over the SBSMA in terms of thermal cracking performance.

The addition of bio-oil or REOB is normally expected to have an obvious softening effect (reduce complex modulus). However, a similar trend was not observed in the δ_8967 kPa_ when incorporating the oil into SBSMA. There was only a slight increase in the δ_8967 kPa_ of both samples with the bio-oil as compared with that of SBS 1 and SBS 2. By contrast, the incorporation of REOB into SBSMA caused a reduction in the figure. Thus, it can be concluded that the utilization of oils would not greatly benefit the SBSMA from the perspective of δ_8967 kPa_ (the resistance to fatigue cracking). A temporary increase in δ_8967 kPa_ was found in the sample (SBS 2–8% REOB) after RTFO. This could be attributed to the SBS degradation caused by oxidation aging, which had a softening effect and temporally increased the phase angle [6].

### 3.4. The Fatigue Life Results from LAS Tests

**Figure 7:**
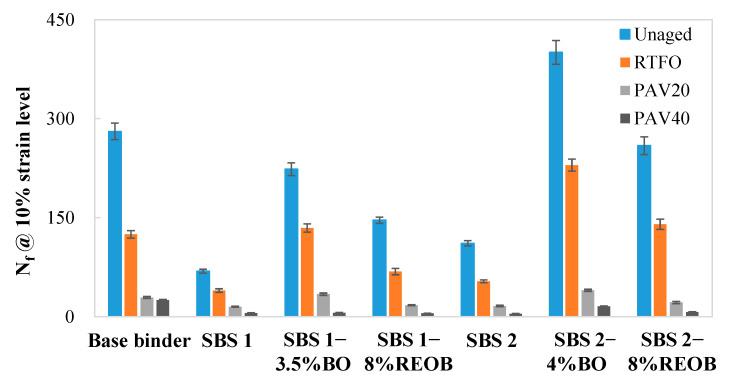
The N_f_ at 10% strain level from LAS tests at all aging conditions.

The N_f_ at a higher strain level (i.e., 10% and above) has been reported to share a stronger correlation with the flexibility index of the semi-circular bending test [21,22]. Thus, in this study, N_f_ at 10% strain was utilized to indicate the resistance to fatigue cracking of the binders, with the results plotted in Figure 7.

First, it is easily found that the SBS modification exhibited an adverse effect on the fatigue cracking performance of the binders, since there was an obvious reduction in N_f_ when comparing the base binder and SBS 1 as well as SBS 2. A significant upward trend was seen in the N_f_ of samples incorporating oils (especially the bio-oil) as compared with SBSMA samples without oils. Additionally, it is worth noting that the N_f_ also varied based on the type of SBS polymer because the N_f_ of SBS 2 is greatly higher than that of SBS 1 and approached the level of the sample SBS 1–8%REOB. Moreover, oxidation aging was found to result in a severely negative effect on all binders in terms of the fatigue cracking performance. A sharp drop in the value of N_f_ was witnessed in all binders after RTFO or PAV20 aging, while the figures of all binders decreased to nearly zero when subjected to 40 h PAV aging.

### 3.5. Correlating Analysis Among Cracking Performance Parameters of Binders

The binder parameters that have been discussed above aimed to describe the benefit of adding oils into SBSMA in terms of the resistance to cracking and depict the degradation of this benefit during the aging process. In fact, these parameters (the indicators of the binder’s rheological behavior) were proposed to estimate and characterize the mixture cracking property. Thus, these parameters inherently share the correlating relationships, to a certain degree. This section focuses on exploring the correlation among binder parameters.

**Figure 8:**
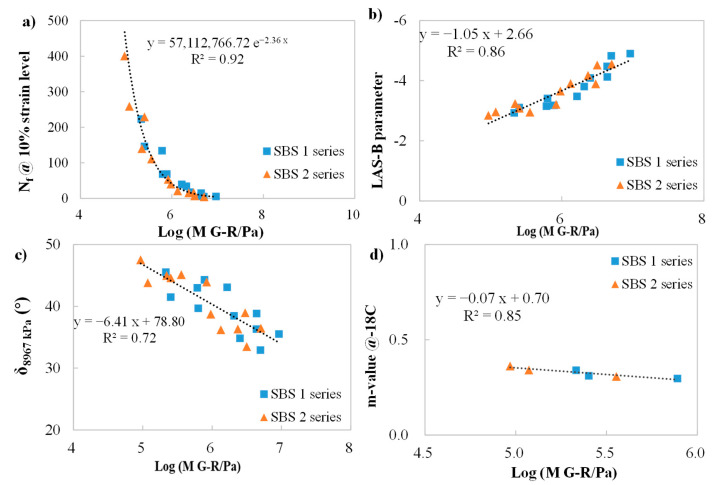
Correlations among cracking performance parameters of binders. (**a**): N_f_ at 10% strain level versus the logarithm of M G–R; (**b**): LAS-B parameter versus the logarithm of M G–R; (**c**): δ_8967 kPa_ versus the logarithm of M G–R; (**d**): m-value versus the logarithm of M G–R.

From Figure 8, strong correlating relationships between Log (M G–R) and other cracking-related indices (not including δ_8967 kPa_) were observed, with the R^2^ ranging from 0.85 to 0.92. All data points could be plotted into one trendline, not showing the material dependency and concluding all aging conditions. Additionally, linear relationships were found in both Log (M G–R) versus LAS-B parameter and Log (M G–R) versus m-value. The N_f_ at 10% strain shared an exponential function law with Log (M G–R), indicating N_f_ could decrease at a faster rate than Log (M G–R) as the aging severity moved forward. The δ_8967 kPa_ evolution during the aging process was also linearly correlated with Log (M G–R), though the correlating degree (R^2^ of 0.72) is not as high as the others.

In conclusion, correlations among cracking-related parameters were found, indicting a consistent trend in parameters during aging. To some extent, one parameter could be utilized to predict the other one.

### 3.6. Correlations Between Chemical and Rheological Indices of Binders

The carbonyl index has been proven to be a semi-quantitative indicator of oxidation aging level. This part aims to discover the relationship between cracking-related parameters and the level of oxidation aging, as measured by the carbonyl index.

Unlike the correlating analysis among cracking-related parameters, which did not exhibit material dependency, the correlations between LAS parameters and the carbonyl index were analyzed separately depending on whether the binder contained bio-oil or not. This could be attributed to the addition of bio-oils that are rich in aliphatic esters in their chemical compositions, which leads to the elevation of the carbonyl index, instead of oxidation aging.

**Figure 9:**
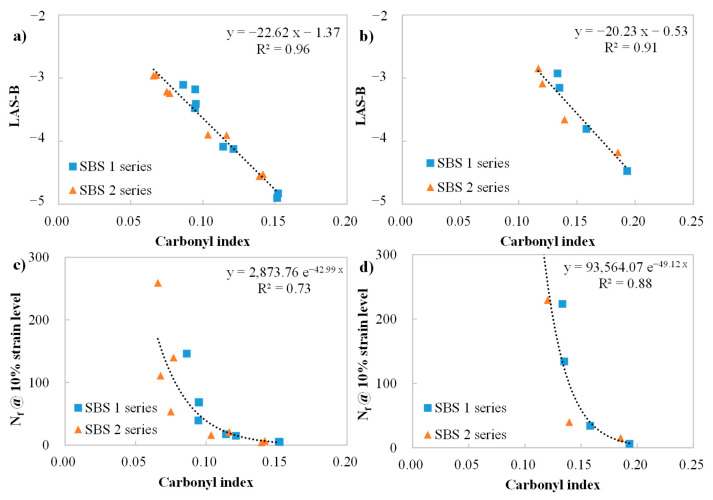
Correlations between the LAS parameters (B and Nf) and carbonyl index: (**a**,**c**) concluding binders without bio-oil; (**b**,**d**) representing binders containing bio-oils.

The effect of oxidation aging on the LAS parameters (plotted in Figure 9) is logical. The LAS-B shared a strong negative linear relationship with aging severity (carbonyl index), with both R^2^ above 0.9. The LAS-B became more negative with aging, revealing that the fatigue life (N_f_) declined faster per unit increase in strain level and denoting the binder became more sensitive to fatigue cracking. The correlations between the carbonyl index and N_f_ are shown in Figure 9c,d; N_f_ decreased as aging increased, following an exponential law, which is consistent with previous findings [33,36].

**Figure 10:**
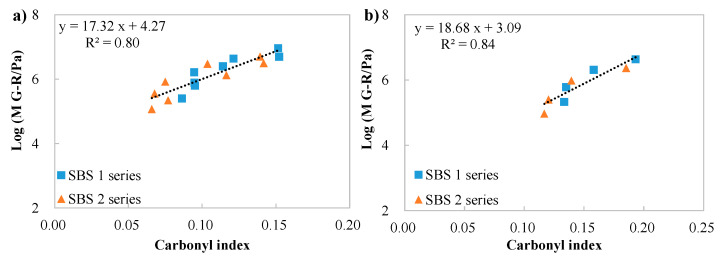
Correlations between the logarithm of M G–R and carbonyl index: (**a**) concluding binders without bio-oil; (**b**) representing binders containing bio-oils.

Comparisons between the carbonyl index and M G–R were also conducted separately, based on whether the binder contained bio-oil or not. The trend depicted in Figure 10 is reasonable, where the logarithm of M G–R increased linearly with the carbonyl index, indicating oxygen-containing substances generated during the aging process led to aging hardening and increased sensitivity to cracking.

**Figure 11:**
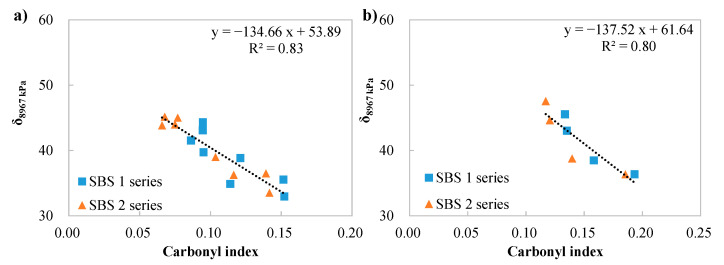
Correlations between the δ_8967 kPa_ and carbonyl index: (**a**) concluding binders without bio-oil; (**b**) representing binders containing bio-oils.

A correlation analysis between the δ_8967 kPa_ (derived from the fatigue factor in PG specification) and the oxidation aging level is shown in Figure 11. The trendline agrees with the findings of the correlations among the LAS parameters and carbonyl index. Aging exhibited an adverse effect on the fatigue life of the binders.

In conclusion, these cracking performance parameters shared high correlations with the carbonyl index, consistently showing a declining trend in the cracking performance as aging severity increased. The addition of bio-oil did not affect the correlating relationship, even though it resulted in material dependency on the correlating analysis. Thus, the cracking performance could be directly predicted by the aging severity of the binders, following the established correlation.

### 3.7. Correlating Analysis Between Mixture and Binder Indices

The purpose of this section is to explore the inherent relationship between the binder and mixture from the perspective of cracking performance. In this correlating analysis, the data were collected from the specimens that had undergone long-term aging, where 20 h PAV aging was conducted on the binders and 8 h oven aging for the mixtures. The CT-index and post-peak slope measured at 25 and 0 °C were utilized to characterize the intermediate- and low-temperature cracking performance, with the results shown in Table 5.

**Figure 12:**
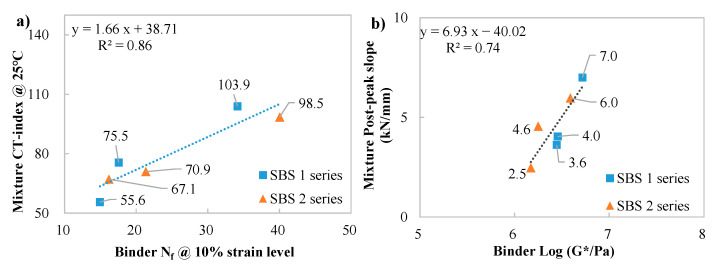
Correlations between intermediate-temp cracking performance of mixture and binder. (**a**): CT-index versus N_f_ at 10% strain level; (**b**): post-peak slope versus the logarithm of complex modulus.

Table From Figure 12a, the correlation between binder N_f_ and mixture CT-index is compelling, indicating the resistance to fatigue cracking of the mixtures is highly controlled by binder property (N_f_). From Figure 12b, it can be seen that the mixture cracking rate was related to the modulus of the binders. This means that stiffer binders could lead to faster cracking in the mixtures.

**Figure 13:**
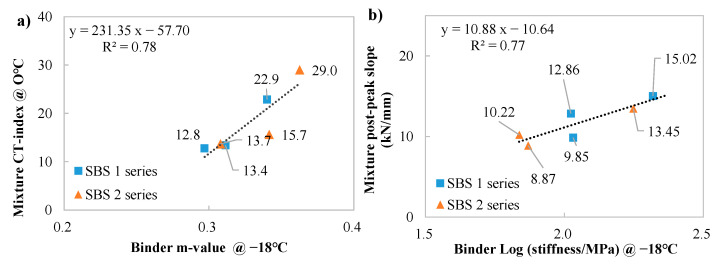
Correlations between low-temp cracking performance of mixture and binder. (**a**): CT-index versus m-value; (**b**): post-peak slope versus the logarithm of stiffness.

The BBR test parameters at −18 °C were compared with the CT-index and post-peak slope at 0 °C to explore the relationship between binder and mixture in terms of thermal cracking performance, as depicted in Figure 13.

A linear trendline was observed in both m-value versus CT-index and the logarithm of stiffness versus post-peak slope. It is known that, at low temperatures, the resistance to cracking of mixtures is also controlled by binder property (stress relaxation property), while the cracking rate is mainly controlled by the binder’s stiffness.

## 4. Conclusions

This work assessed the benefits of combining softening oils with SBSMA in terms of cracking properties, in which various rheological indices and the carbonyl index were utilized to track and evaluate these benefits throughout the aging process. The inherent relationships among the measured binder indices were carefully analyzed, while the correlations of cracking-performance parameters between binder and mixture were also well conducted. The definitions of abbreviations are shown in Table 6. The detailed findings are summarized as follows:

(1) The stiffness and m-value results show that LT cracking performance was not greatly improved by the SBS modification, whereas the application of softening oils into SBSMA would greatly enhance the LT cracking properties. Additionally, the OMI results demonstrated that bio-oil highly outperformed REOB: bio-oil exhibited four to six times better efficiency than that of REOB with regard to improving the stress relaxation property.

(2) From the black diagrams, the addition of softening oils into SBMA had a significant effect on improving cracking properties: shifting the curve downwards in the graph. By contrast, the SBS modification and aging had adverse effects, both of which caused the curve to move from the bottom-right to the upper-left (approaching cracking). Bio-oil was found to be capable of not only reducing the complex modulus (softening effect) but also enhancing the viscous response (phase angle), whereas REOB only exhibited the softening effect.

(3) The δ_8967 kPa_ result shows that the fatigue life of SBSMA is not greatly improved by the softening oils. On the other hand, the N_f_ at 10% strain level (obtained from LAS) results confirm the benefit of softening oils in terms of resistance to fatigue cracking. The fatigue life of SBSMA also varies depending on the type of SBS polymers and oils. However, the benefit brought about by the softening oils on the fatigue life diminishes after long-term aging, since the N_f_ of all samples at PAV40 is close (near zero).

(4) The results of correlations among cracking-performance parameters of the binders indicate that N_f_, LAS-B, and m-value share a strong relationship with the logarithm of M G–R, with R^2^ above 0.85, also displaying a consistent trend during the aging process. In correlating, all data points could be plotted into the one trendline, not showing material dependency. Thus, one parameter, like M G–R, could be utilized to predict the other cracking-performance parameter.

(5) A declining trend in cracking-performance parameters is observed with the oxidation aging level (carbonyl index), but correlating analysis needs to be performed separately based on the application of bio-oil or not, indicating material dependency. This is due to the chemical composition of bio-oil that is rich in aliphatic esters, leading to the increase in the carbonyl index, not by oxidation aging.

(6) Correlations between binders and mixtures show that the resistance to fatigue cracking of mixtures is highly controlled by the binder property (N_f_); meanwhile, the mixture cracking rate is related to the modulus of binders (G*). At low temperatures, mixture cracking performance is highly related to the binder’s relaxation property (m-value), while the cracking rate is primarily controlled by the binder’s stiffness.

**Table 6:** List of abbreviations.

Abbreviations	Definitions
SBS	Styrene–butadiene–styrene
SBSMA	Styrene–butadiene–styrene-modified asphalt
Bio-oil	Bio-based oil
REOB	Re-refined engine oil bottom
M G–R	Modified Glover–Rowe parameter
G*	Complex modulus
δ	Phase angle
δ_8967 kPa_	δ at G* = 8967 kPa
LAS	Linear amplitude sweep
N_f_	Fatigue life obtained from LAS
IDEAL-CT	Indirect tensile asphalt cracking test
FTIR	Fourier transform infrared spectroscopy
PG	Performance grade
HT PG	High-temperature performance grade
LT PG	Low-temperature performance grade
VECD	Viscoelastic continuum damage
AC	Asphalt concrete
RTFO	Rolling thin film oven
PAV	Pressure aging vessel
PAV20/40	20/40 h PAV
BBR	Bending beam rheometer
SENB	Single edge notched beam
G_f_	Fracture energy
OMI	Oil modification index

## Figures and Tables

**Table 1:** Fundamental properties of bio-oil and REOB.

Properties	Bio-Oil	REOB	Standard
Acid value (mg KOH/g)	23	35	ASTM D 1980–87 [27]
Flash point (°C)	>290	220	AOCS Cc 9a-48 [28]
Density @ 25 °C (g/cm^3^)	1.03	0.94	ASTM D1475 [29]
Viscosity @ 60 °C (mPa·s)	27.5	305	ASTM D4402 [30]

**Table 2:** The compositions and descriptions of binder samples.

	SBS1	SBS1–3.5%BO	SBS1–8%REOB	SBS2	SBS2–4%BO	SBS2–8%REOB
Base binder	96.9%	93.4%	88.9%	95.4%	91.4%	87.4%
Dushanzi	3.0%	3.0%	3.0%	0.0%	0.0%	0.0%
LG 501	0%	0%	0%	4.5%	4.5%	4.5%
Bio-oil	0%	3.5%	0%	0%	4.0%	0%
REOB	0%	0%	8.0%	0%	0%	8.0%
Sulfur	0.1%	0.1%	0.1%	0.1%	0.1%	0.1%
HT PG	82.9	76.2	77.1	82.7	75.7	77.8
LT PG	−27.6	−34.3	−30.0	−29.2	−34.1	−34.1
PG	82–28	76–34	76–28	82–28	76–34	76–34

**Table 3:** The information of mix design.

Gradation	Upper Limit	Lower Limit	The Median	Design Mix
	Passing Percent (%)			
16 mm	100	100	100	100
13.2 mm	100	90	95	95.2
9.5 mm	85	68	76.5	72.1
4.75 mm	68	38	53	42.5
2.36 mm	50	24	37	27.9
1.18 mm	38	15	26.5	19.1
0.6 mm	28	10	19	14.1
0.3 mm	20	7	13.5	10.2
0.15 mm	15	5	10	8.5
0.075 mm	8	4	6	6.2
Binder content (%)	NA	NA	NA	5.2

**Table 4:** OMI results of bio-oil and REOB.

Samples		OMI	
		Log Stiffness (MPa/%)	m-Value (/%)
SBS1	Bio-oil	−0.08	0.012
	REOB	−0.04	0.002
SBS2	Bio-oil	−0.09	0.014
	REOB	−0.05	0.004

**Table 5:** Results of CT-index and post-peak slope obtained from mixture cracking tests at 25 and 0 °C.

Samples	Oil Type	25 °C		0 °C	
		CT-Index	Post-Peak Slope (KN/mm)	CT-Index	Post-Peak Slope (KN/mm)
SBS1	NA	55.6	7.0	12.8	15.0
	Bio-oil	103.9	3.6	22.9	9.9
	REOB	75.5	4.0	13.4	12.9
SBS2	NA	67.1	6.0	13.7	13.5
	Bio-oil	98.5	2.5	29.0	8.9
	REOB	70.9	4.6	15.7	10.2

## Data Availability

The original contributions presented in this study are included in the article. Further inquiries can be directed to the corresponding author.
